# gga-miR-155 Enhances Type I Interferon Expression and Suppresses Infectious Burse Disease Virus Replication via Targeting SOCS1 and TANK

**DOI:** 10.3389/fcimb.2018.00055

**Published:** 2018-03-07

**Authors:** Bin Wang, Mengjiao Fu, Yanan Liu, Yongqiang Wang, Xiaoqi Li, Hong Cao, Shijun J. Zheng

**Affiliations:** ^1^State Key Laboratory of Agrobiotechnology and College of Veterinary Medicine, China Agricultural University, Beijing, China; ^2^Key Laboratory of Animal Epidemiology of the Ministry of Agriculture, China Agricultural University, Beijing, China; ^3^Department of Preventive Veterinary Medicine, College of Veterinary Medicine, China Agricultural University, Beijing, China

**Keywords:** microRNA, type I IFN, IBDV, SOCS, TANK

## Abstract

Infectious bursal disease (IBD) is an acute, highly contagious, and immunosuppressive avian disease caused by IBD virus (IBDV). MicroRNAs (miRNAs) are involved in host-pathogen interactions and innate immune response to viral infection. However, the role of miRNAs in host response to IBDV infection is not clear. We report here that gga-miR-155 acts as an anti-virus host factor inhibiting IBDV replication. We found that transfection of DF-1 cells with gga-miR-155 suppressed IBDV replication, while blockage of the endogenous gga-miR-155 by inhibitors enhanced IBDV replication. Furthermore, our data showed that gga-miR-155 enhanced the expression of type I interferon in DF-1 cells post IBDV infection. Importantly, we found that gga-miR-155 enhanced type I interferon expression via targeting SOCS1 and TANK, two negative regulators of type I IFN signaling. These results indicate that gga-miR-155 plays a critical role in cell response to IBDV infection.

## Introduction

Infectious bursal disease (IBD), also called Gumboro disease, is an acute, highly contagious disease in young chickens that is considered to be one of the major infectious diseases threatening the poultry industry (Pitcovski et al., 2003). Its causative agent, infectious bursal disease virus (IBDV), destroys the B-lymphocyte precursors and causes a high degree of immunosuppression. The surviving chickens suffer from a severe immunosuppression leading to an increased susceptibility to other pathogens (Stricker et al., 2010). IBDV belongs to the genus *avibirnavirus* of the family *birnaviridae*, which consists of two segments of double-stranded RNA (A and B; Azad et al., 1985; Tacken et al., 2004). Segment B (2.8 kb) encodes VP1 (90 kDa) (Morgan et al., 1988), an RNA-dependent RNA polymerase (Tacken et al., 2004; Pan et al., 2009). Segment A contains two partially overlapping open reading frames (ORFs) (Kibenge et al., 1991). The first ORF encodes the nonstructural viral protein vp5 (17 kDa), while the second one encodes an approximate 110 kDa polyprotein precursor that can be cleaved by viral protease VP4 to form viral proteins VP2 (41 kDa), VP3 (32 kDa), and VP4 (24 kDa) (Azad et al., 1985; Hudson et al., 1986; Jagadish et al., 1988; Kibenge et al., 1991).

miRNAs are a family of small, non-coding RNAs, ranging in length from 18 to 25 nucleotides, which mediate either degradation of RNA or translational suppression by binding to complementary sites primarily in the 3′UTRs of mRNAs (He and Hannon, 2004), playing a wide variety of roles in cancer (Lewis et al., 2005), inflammation (O'Connell et al., 2012), and immune responses (Baltimore et al., 2008). Experimental evidence indicated the complicated interaction between miRNAs and viruses (Tong et al., 2013; Dai et al., 2015). On one hand, viruses may encode their own miRNA or utilize host miRNA to help in the viral life cycles and other aspects of pathogenesis (Hu et al., 2015). On the other hand, cellular miRNAs can directly target viral RNAs during infections or regulate various aspects of innate and adaptive immune responses to inhibit virus replication (Wang et al., 2010; Zhang et al., 2014).

The pattern recognition receptors (PRRs) in innate immune system, such as Toll-like receptors (TLRs) and RIG-I-like receptors (RLRs), can recognize viral pathogen associated molecular patterns (PAMPs) and recruit downstream kinases that phosphorylate downstream adaptor proteins to transduce cell response signaling leading to the translocations of transcriptional regulators NF-κB, IRF3/7 and/or AP-1 to initiate the expression of type I interferon. It has been reported that chicken type I interferon has strong antiviral activity in IBDV-infected cells (O'Neill et al., 2010; Cai et al., 2012), suggesting that chicken type I interferon of host cells plays a critical role in combating IBDV. Recent evidence show that host miRNAs are involved in modulation of host antiviral activities via induction of type I IFNs (Li et al., 2015; Chen et al., 2016).

Although IBDV has been one of the most studied avian viruses, few reports are available regarding the effects of host miRNAs on IBDV infection and its underlying molecular mechanisms. It was reported that gga-miR-21 suppressed IBDV replication through targeting viral genomic RNA (Wang et al., 2013), but the exact mechanism of such suppression is still unclear. In this study, we screened IBDV-infected DF-1 cells (immortal chicken embryo fibroblast) for the potential host miRNAs regulating host response against IBDV infection. We found that gga-miR-155 effectively suppressed IBDV replication by inducing IFN-β expression via targeting SOCS1 and TANK, two negative regulators for interferon expressions (Dai et al., 2006; Kawagoe et al., 2009; Fujimoto and Naka, 2010; Huang et al., 2015). Thus, gga-miR-155 plays a critical role in cell response to IBDV infection.

## Materials and methods

### Cells and virus

DF-1 cells (immortal chicken embryo fibroblast) were obtained from ATCC. Cells were cultured in Dulbecco modified Eagle medium (DMEM) (Thermo Fisher, USA) supplemented with 10% fetal bovine serum (FBS) in a 5% CO2 incubator. *Lx*, a cell culture-adapted IBDV strain, was kindly provided by Jue Liu (Beijing Academy of Agriculture and Forestry, Beijing, China).

### Reagents

PGL3-Control vector was kindly provided by Wenhai Feng (China Agricultural University, Beijing, China). Restriction enzyme XbaI was purchased from TaKaRa, Poly(I:C) from Sigma, and HRP-conjugated goat anti-mouse IgG and fluorescein isothiocyanate (FITC)-conjugated goat anti-mouse IgG from Ding-Guo (China). Anti-VP3 monoclonal antibody and anti-TANK polyclonal antibodies were developed in our laboratory. All miRNA mimics and inhibitors (chemically-modified and single-stranded RNA oligonucleotide that is reverse complement sequence of mature gga-miR-155) were synthesized by GenePharma Company (Shanghai, China).

### Deep sequencing and miRNA target prediction

Deep sequencing was performed by LC Sciences (Hangzhou, China) on DF-1 cells infected with mock or IBDV at an MOI of 1 for 24 h (accession number of deep sequencing in GEO database: GSE90095). miRNA targets in host cells were predicted by TargetScan (version 3.1; Whitehead Institude for Biomedical Research [http://www.targetscan.org/mamm_31/]) and miRanda [August 2010 Release; Memorial Sloan Kettering Cancer Center (http://www.microrna.org/microrna/home.do)].

### Transfection of miRNA mimics or inhibitors for the measurement of IBDV growth

DF-1 cells were seeded in 24-well or 12-well plates for 24 h, and then miRNA mimics or inhibitors (200 nM) were transfected using Lipofectamine 2000 transfection reagents (invitrogen). Twelve hours after transfection, cells were infected with IBDV. Twenty-four hours after IBDV infection, cell cultures were collected for virus titration or RNA quantification, or cells were fixed for indirect immunofluorescent antibody assay.

### Measurement of IBDV growth in DF-1 cells

Normal cells or cells receiving miRNA mimics or mimic controls were infected with IBDV at an MOI of 0.01. Twenty-four hours after infection, cell cultures were freeze-thawed three times and centrifuged at 2,000 × g for 10 min. The viral contents in the supernatants were titrated using 50% tissue culture infective doses (TCID50) in DF-1 cells. Briefly, the viral solution was diluted by 10-fold in DMEM. A 100 μl aliquot of each diluted samples was added to the wells of 96-well plates, followed by addition of 100 ul of DF-1 cells at a density of 5 × 10^5^ cells/ml. Cells were cultured for 7 days at 37°C in 5% CO2. Tissue culture wells with a cytopathic effect (CPE) were determined as positive. The titer was calculated on the basis of a previously described method (Reed and Muench, 1938).

### Indirect immunofluorescence antibody assay (IFA)

DF-1 cells were transfected with miRNA mimics or miRNA controls for 12 h at the concentration of 200 nM, and followed by infection with IBDV *Lx* strain at an MOI of 0.01 for 24 h. The cells were fixed with 4% paraformaldehyde, permeabilized with 0.2% Triton X-100, blocked with 1% bovine serum albumin, and probed with mouse anti-VP3 monoclonal antibody, followed by incubation with FITC-conjugated goat anti-mouse antibody (green). Cells were examined with a fluorescence microscope (OLYMPUS 1X71; Nikon, Japan).

### RNA isolation and quantitative real-time PCR (qRT-PCR) analysis

Total RNA and miRNA were prepared from DF-1 cells using EASYspin Plus kit or RNA mini kit (aidlab Biotechnology, China) per the manufacturer's instructions. mRNAs were reversely transcribed with primescript^TM^ RT Reagent kit (Takara). Quantitative RT-PCR analysis was performed using Tli RnaseH Plus (Takara) on LightCycler 480II (Roche, USA). Specific primers for chicken IFN-α (chIFN-α) (5′-CCA GCA CCT CGA GCA AT-3′ and 5′-GGC GCT GTA ATC GTT GTC T-3′), chicken IFN-β (chIFN-β) (5′-GCC TCC AGC TCC TTC AGA ATA CG-3′ and 5′-CTG GAT CTG GTT GAG GAG GCT GT-3′), chicken IRF3 (chIF3) (5′-GCT CTC TGA CTC TTT CAA CCT CTT-3′ and 5′-AAT GCT GCT CTT TTC TCC TCT G-3′), and chicken GAPDH (5′-TGC CAT CAC AGC CAC ACA GAA G-3′ and 5′-ACT TTC CCC ACA GCC TTA GCA G-3′) were designed with reference to previous publications (Li et al., 2007; Abdul-Careem et al., 2008; Liu et al., 2010). Specific primers for chicken SOCS1 (5′- CTC GCA AGC GGA TTT CAG TAG-3′, 5′- GGG CTC AGA CTT CAG CTT CTC-3′) were designed and synthesized by Sangon Biotech (Shanghai, China). GAPDH gene was utilized as the reference gene. All quantitative real-time PCR experiments were performed in triplicate. The PCR was performed in a 20 μl volume containing 1 μl of cDNA, 10 μl of 2 × SYBR green Premix *Ex Taq* (TaKaRa), and a 0.4 μM of each gene-specific primers. Thermal cycling parameters were as follows: 94°C for 2 min; 40 cycles of 94°C for 20 s, 55°C for 20 s, and 72°C for 20 s; and 1 cycle of 95°C for 30 s, 60°C for 30 s, and 95°C for 30 s. The final step was to obtain a melt curve for the PCR products to determine the specificity of the amplification. qRT-PCR analysis of gga-miR-155 was performed with RT-PCR Quantitation Kit (GenePharma, China).

### Construction of luciferase reporters and luciferase activity assays

A 349 bp fragment of SOCS1 gene or a 480 bp fragment of TANK gene around the predicted gga-miR-155 target sites were amplified and inserted into the site downstream of the firefly luciferase gene in the pGL3-Control vector at the XbaI site to create the wild type 3′UTR vector. Both miRNA seed sites mutant in SOCS1 gene or TANK gene were made by mutating the underlined 4 nucleotides in the 8mer seed sequences (SOCS1: 5′-TCA GAT CTA AGT ACA GC ATTA A-3′ mutated to 5′-TCA GAT CTA AGT ACA GC TAAT A. The first seed sequence of TANK: 5′-TGC TAA TAT ACC TTT CCA GC ATTA ATCA-3′ mutated to 5′-TGC TAA TAT ACC TTT CCA GC TAAT ATCA-3′, and the second seed sequence of TANK: 5′-TGG TTT TTG ATA AAA TTA GC ATTA ATAT-3′ mutated to 5′-TGG TTT TTG ATA AAA TTA GC TAAT ATAT-3′) using a fast mutagenesis system (Transgene, China) per the manufacturer's instructions. Another vector pRL-TK containing the Renilla luciferase was used as a control.

### Western blot analysis

Chicken *socs1* gene was cloned from DF-1 cells using the specific primes 5′- CGC GGA TCC ATG GTA GCG CAC AGC AAG GTG-3′ (sense), and 5′- ACG CGT CGA CG TTA GAT CTG AAA CGG GAA GGA-3′ (anti-sense) with reference to the sequence in GenBank (accession no.NM_001137648.1). Chicken socs1 gene was then subcloned into pET-28a vector. The pET-28a-socs1 recombinant plasmid was used to transform *E. coli* BL21, and SOCS1-his recombinant protein was expressed at 37°C and purified by a protein purification kit (Ni Sepharose™ 6 Fast Flow, GE Company, USA) per the manufacturer's instructions. To develop polyclonal antibody against SOCS1, BALB/c mice were immunized with SOCS1-his recombinant protein. After immunization, anti-SOCS1 polyclonal antibody was obtained from the sera of BALB/c mice vaccinated with SOCS1-his recombinant protein. For detection of TANK in DF-1 cells, cell lysates were prepared using a non-denaturing lysis buffer (50 nM Tris-HCl, pH 8.0, 150 nM NaCl, 1% TritonX-100, 5 nM EDTA, 10% glycerol, 10 nM dithiothreitol, 1 × complete cocktail protease inhibitor). The cell lysates were boiled with 6 × SDS loading buffer for 10 min and fractionated by electrophoresis on 12% SDS-polyacrylamide gels, and resolved proteins were transferred onto polyvinylidene difluoride (PVDF) membranes. After blocking with 5% skimmed milk, the membranes were incubated with anti-TANK or anti-GAPDH antibody, followed by HRP-conjugated anti-Mouse secondary antibody. Blots were developed using an enhanced chemiluminesence (ECL) kit per the manufacturer's instruction.

### Knockdown of TANK by RNA interference (RNAi)

The siRNA was designed by GenePharma Company (Shanghai, China) and used to knockdown TANK in DF-1 cells. The siRNAs for targeting TANK in DF-1 cells included the followings: RNAi#1 (sense, 5′-GGA CCA UGC UGU GAA AGA ATT-3′; antisense, 5′-UUC UUU CAC AGC AUG GUC CTT-3′), RNAi#2 (sense, 5′-CCA GAU AAG CCU GAA UUA UTT-3′; antisense, 5′-AUA AUU CAG GCU UAU CUG GTT-3′), RNAi#3 (sense, 5′-GCU GUU AAC CUC AGU AAA UTT-3′; antisense, 5′-AUU UAC UGA GGU UAA CAG CTT-3′), and negative control (sense, 5′-UUC UCC GAA CGU GUC ACG UTT-3′; antisense, 5′-ACG UGA CAC GUU CGG AGA ATT-3′). DF-1 cells were seeded on 12-well or 24-well plates. Twenty-four hours after culture, cells were transfected with siRNA using RNAiMAX per the manufacturer's instructions (Invitrogen). Double transfections were performed at a 24 h interval. Twenty-four hours after the second transfection, cells were harvested for further analysis.

### Statistical analysis

The significance of the differences between gga-miR-155-transfected cells and controls in gene expression, and viral growth was determined by the Mann-Whitney test and analysis of variance (ANOVA) accordingly.

## Results

### gga-miR-155 inhibits IBDV replication

**Figure 1:**
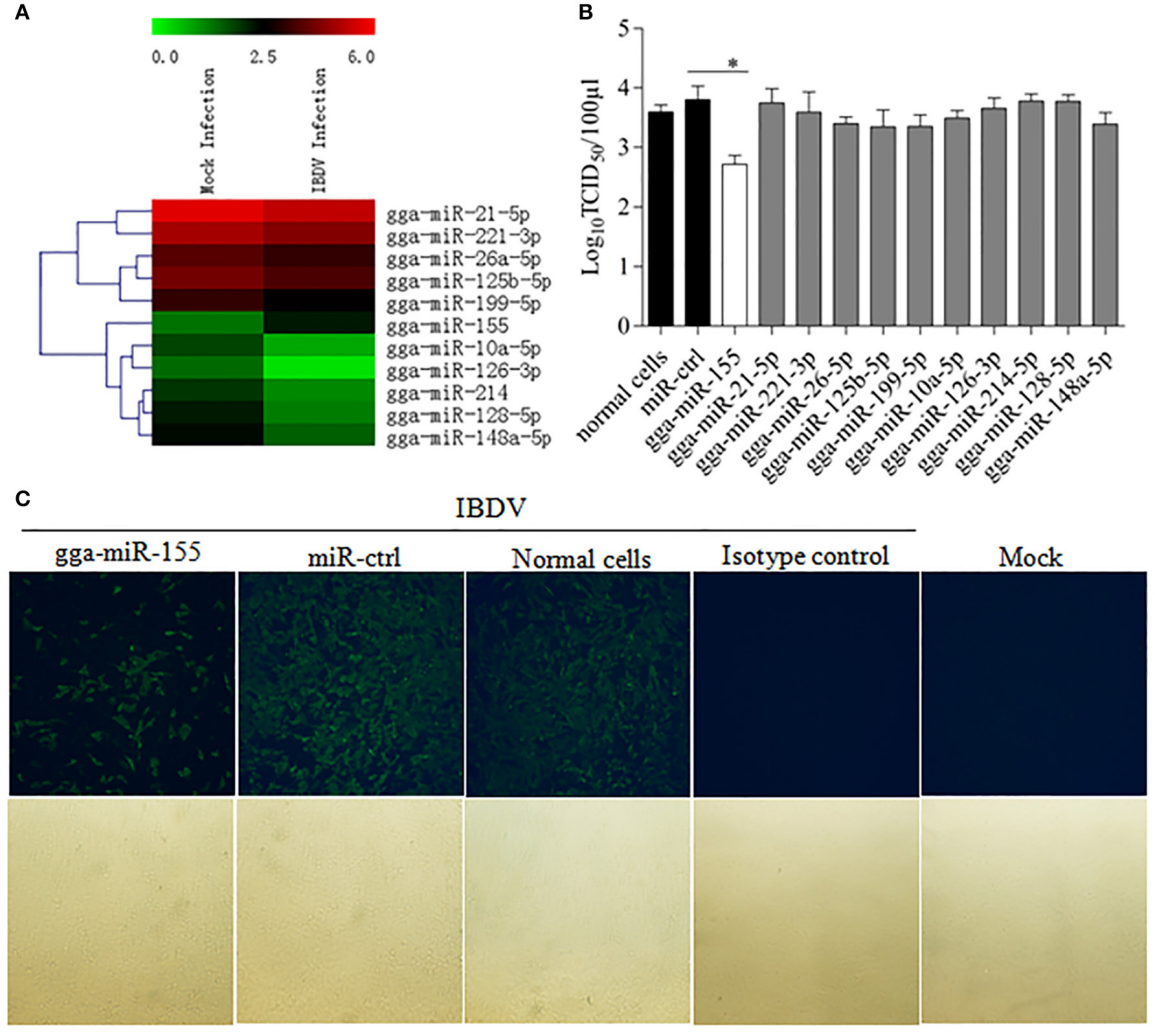
Examination of miRNAs in DF-1 cells with or without IBDV infection. **(A)** The heat map illustrates the expression profiles of 11 miRNAs in DF-1 cells infected with or without IBDV in deep sequencing. Each row represents the logarithms (base 10) of the expression level of one single miRNA. The green color stands for the decreased expressions of miRNAs while the red for increased expressions of miRNAs. **(B,C)** gga-miR-155 inhibits IBDV replication. DF-1 cells were transfected with the mimics of indicated miRNAs or miRNA control for 12 h at the concentration of 200 nM, and followed by infection with IBDV *Lx* strain at an MOI of 0.01 for 24 h. Viral titers in the cell cultures were determined by TCID_50_ using DF-1 cells **(B)**, and IBDV VP3 protein was examined by immunofluorescent antibody assay (IFA) **(C)**. The pictures in the upper panels in **(C)** were taken under a fluorescent microscope, and those in the lower panels under a light microscope at 100 × Magnification. Results are representative of three independent experiments and presented as means ± SD. ^*^p < 0.05.

To identify the miRNA expressed by host cells infected by IBDV, we performed Deep sequencing to identify the miRNA expressions in DF-1 cells infected with IBDV *Lx* strain at an MOI of 1 for 24 h. Among the differentially expressed miRNAs, 369 miRNAs were significantly upregulated and 169 downregulated (Figure 1A; Data Sheet 1). To examine the biological relevance of differentially expressed miRNAs during IBDV infection, we selected 11 miRNAs that were sufficiently expressed in IBDV-infected cells, and transfected DF-1 cells with these miRNAs mimics to examine their effects on IBDV replication. As a result, among the 11 miRNAs candidates, gga-miR-155 partially inhibited IBDV replication in cells compared with miRNA controls (Figure 1B). Consistently, IBDV VP3 protein expressions in IBDV-infected DF-1 cells were also inhibited by gga-miR-155 transfection as demonstrated by the IFA assay using monoclonal antibody to VP3 (Figure 1C). These data suggest that gga-miR-155 inhibits IBDV replication.

### gga-miR-155 expression is upregulated during IBDV infection

**Figure 2:**
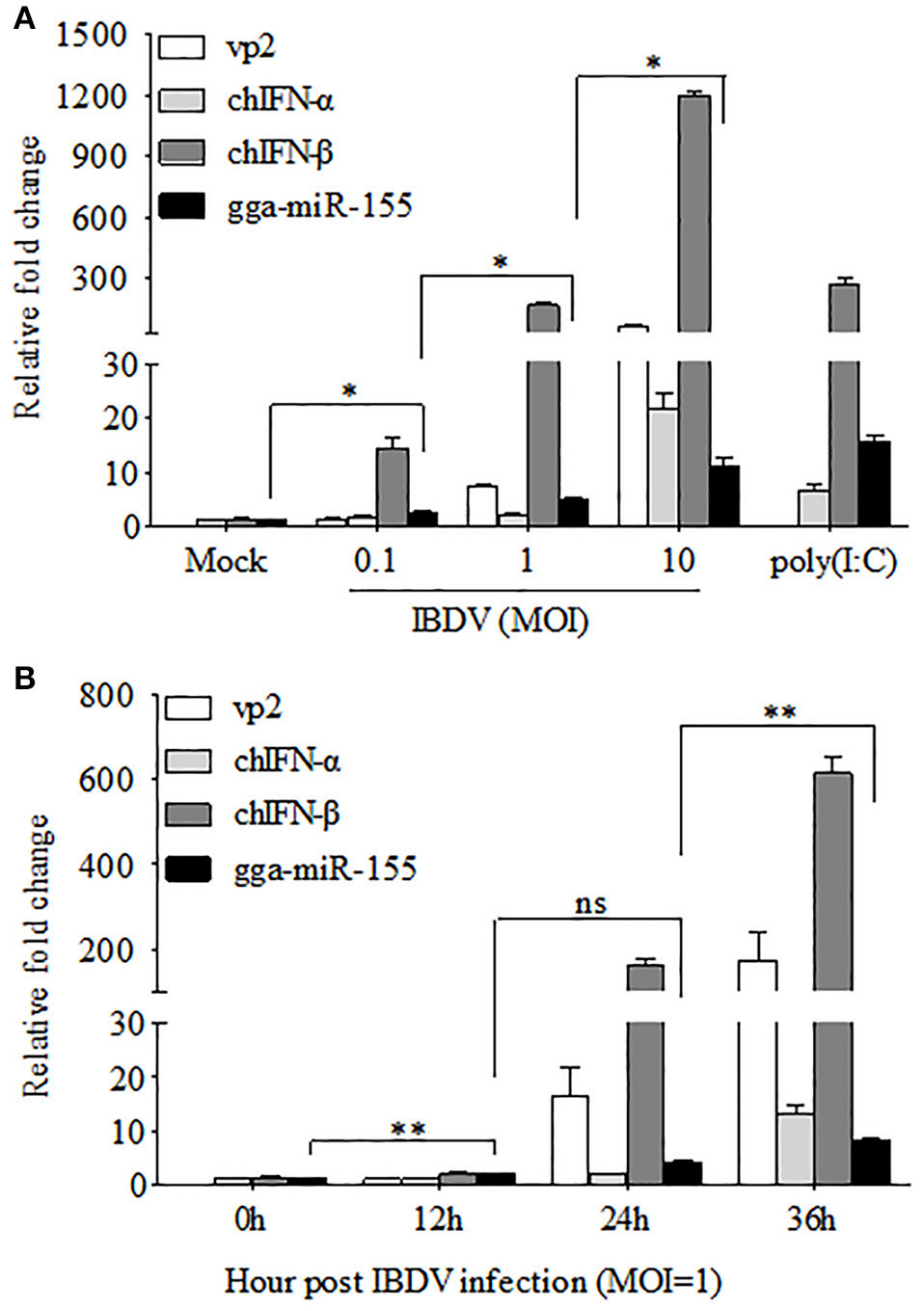
gga-miR-155 expression was upregulated during IBDV infection. **(A)** DF-1 cells were infected with IBDV (at an MOI of 0.1, 1, or 10) or mock, or transfected with poly(I:C). Twenty-four hours after infection or transfection, total RNA was extracted and qRT-PCR was performed to detect IBDV vp2, chIFN-α, chIFN-β, and gga-miR-155 transcripts. **(B)** DF-1 cells were infected with IBDV at an MOI of 1. Total RNA was extracted and qRT-PCR was performed to detect IBDV vp2, chIFN-α, chIFN-β, and gga-miR-155 transcripts at different time points (0, 12, 24, and 36 h). The relative level of vp2, chIFN-α, chIFN-β, or gga-miR-155 mRNA expression is calculated as follows: the mRNA expression of vp2, chIFN-α, chIFN-β, or gga-miR-155 in IBDV-infected or poly(I:C)-simulated cells/ that of the normal cells. GAPDH and U6 were used as internal controls. Data are representative of three independent experiments and presented as means ± SD. ^**^p < 0.01 and ^*^p < 0.05.

The fact that poly(I:C) induced upregulation of miR-155 in different cell lines prompted us to investigate the possibility that upregulation of gga-miR-155 in IBDV-infected DF-1 cells is related to type I IFN signaling (O'Connell et al., 2007; Wang et al., 2010; Zhang et al., 2012). With poly(I:C) as positive control triggering type I IFN expression, we found that both gga-miR-155 and type I IFN were upregulated in DF-1 cells infected with different MOIs of IBDV in a dose-dependent manner (Figure 2A). Furthermore, gga-miR-155 and type I IFN expressions were also upregualted in cells with IBDV infection in a time-dependent manner (Figure 2B). These results suggest that type I IFN signaling is highly correlated to gga-miR-155 expression in IBDV-infected cells (Image 1).

### Inhibition of endogenous gga-miR-155 enhances IBDV infection

**Figure 3:**
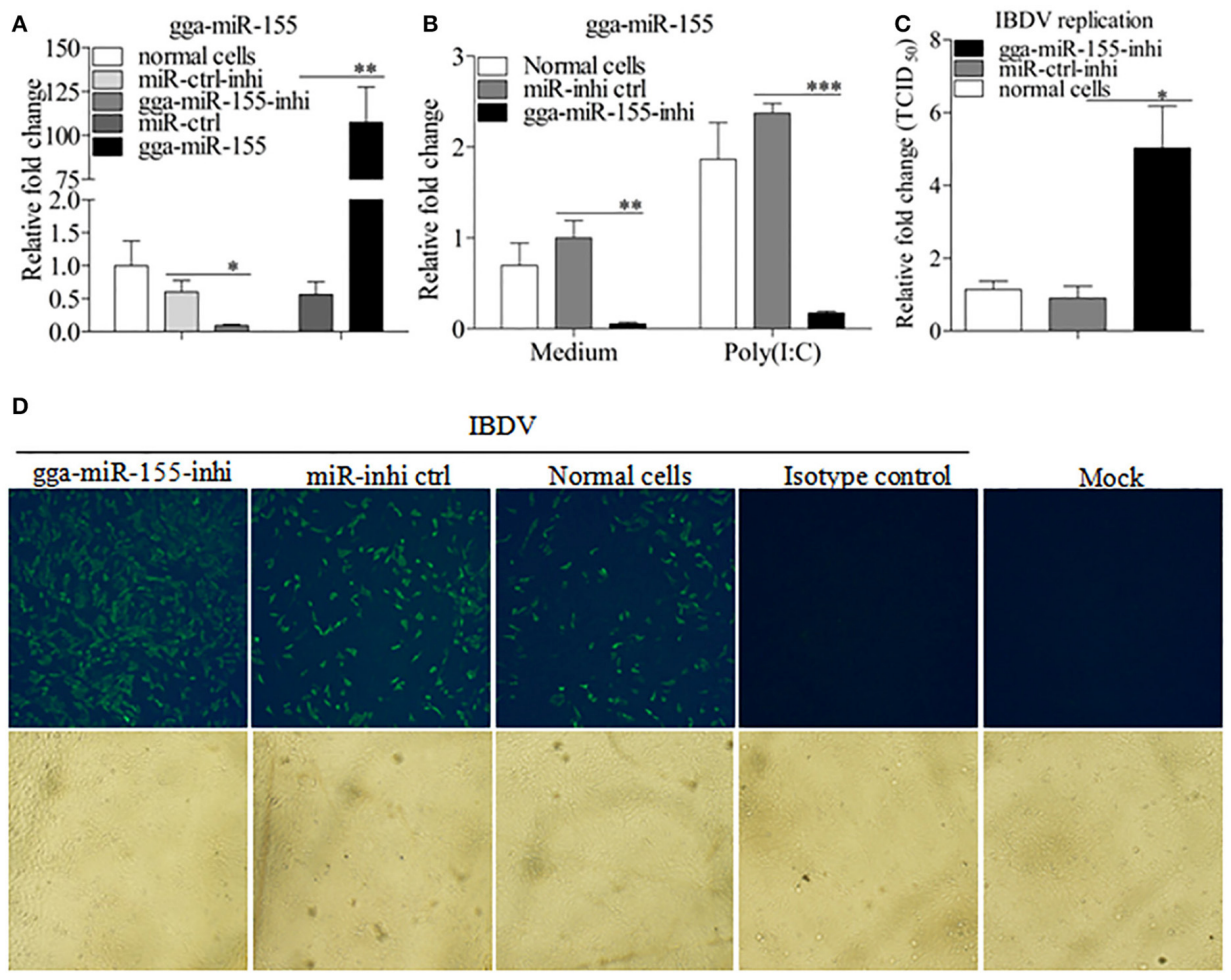
Inhibition of endogenous gga-miR-155 enhances IBDV infection. **(A)** Measurement of gga-miR-155 expressions in cells transfected with gga-miR-155 or gga-miR-155-inhibitor. DF-1 cells were transfected with gga-miR-155, miRNA control, gga-miR-155-inhibitor, or miRNA-inhibitor control. Twelve hours post-transfection, cells were harvested for quantifying the expression of gga-miR-155 by qRT-PCR. The relative level of gga-miR-155 expression in DF-1 cells is calculated as follows: gga-miR-155 expression of miRNA transfected cell sample/ gga-miR-155 expression of the normal cells. U6 was used as an internal control. Data are representative of three independent experiments and presented as means ± SD. ^**^p < 0.01 and ^*^p < 0.05. **(B)** DF-1 cells were transfected with gga-miR-155-inhibitor mimics or miRNA-inhibitor control at the concentration of 200 nM. Twenty-four hours after transfection, cells were treated with Poly(I:C) at a final concentration of 2 μg/ml. Twelve hours after Poly(I:C) treatment, cells were harvested for quantifying the expression of gga-miR-155 by qRT-PCR. The relative level of gga-miR-155 expression is calculated as follows: the mRNA expression of gga-miR-155 in gga-miR-155 inhibitor treated cells or normal cells treated with Poly(I:C)/that of miRNA inhibitor control treated cells in medium control. U6 was used as an internal control (C&D) Inhibition of endogenous gga-miR-155 enhances IBDV infection. DF-1 cells were transfected with gga-miR-155 inhibitor or miRNA inhibitor control for 12 h at the concentration of 200 nM, followed by infection with IBDV *Lx* strain at an MOI of 0.01. Twenty-four hours after IBDV infection, cell cultures were collected for titration of virus by TCID_50_ **(C)**, or cells were fixed for detection of IBDV VP3 protein by immunofluorescent antibody assay **(D)**. The pictures in the upper panels in (D) were taken under a fluorescent microscope, and these in the lower panel under a light microscope at 100 × Magnification. Data are representative of three independent experiments and presented as mean ± SD. ^***^p < 0.001, ^**^p < 0.01, and ^*^p < 0.05.

To further examine the effects of gga-miR-155 on IBDV replication, we knocked down the expression of endogenous gga-miR-155 in DF-1 cells using inhibitors and infected these cells with IBDV. As shown in Figure 3A, gga-miR-155 inhibitor could effectively knockdown endogenous gga-miR-155 expression, reducing gga-miR-155 expression by around 6 folds as compared to that of the inhibitor controls (*p* < 0.05). Similarly, gga-miR-155 inhibitor also markedly knocked down the expression of endogenous gga-miR-155 by about 13 folds relative to the inhibitor controls in cells with poly(I:C) treatment (Figure 3B). Importantly, knockdown of gga-miR-155 by inhibitors markedly facilitated IBDV replication as demonstrated by viral titration using TCID_50_ and viral protein VP3 expression using IFA assay, and the increase of viral yields was about 5 folds as determined by TCID_50_ assay (Figures 3C,D). These results clearly establish the role of gga-miR-155 as an anti-viral factor in cell response to IBDV infection.

### gga-miR-155 enhanced IBDV-induced expressions of chicken type-I interferon and chIRF3

**Figure 4:**
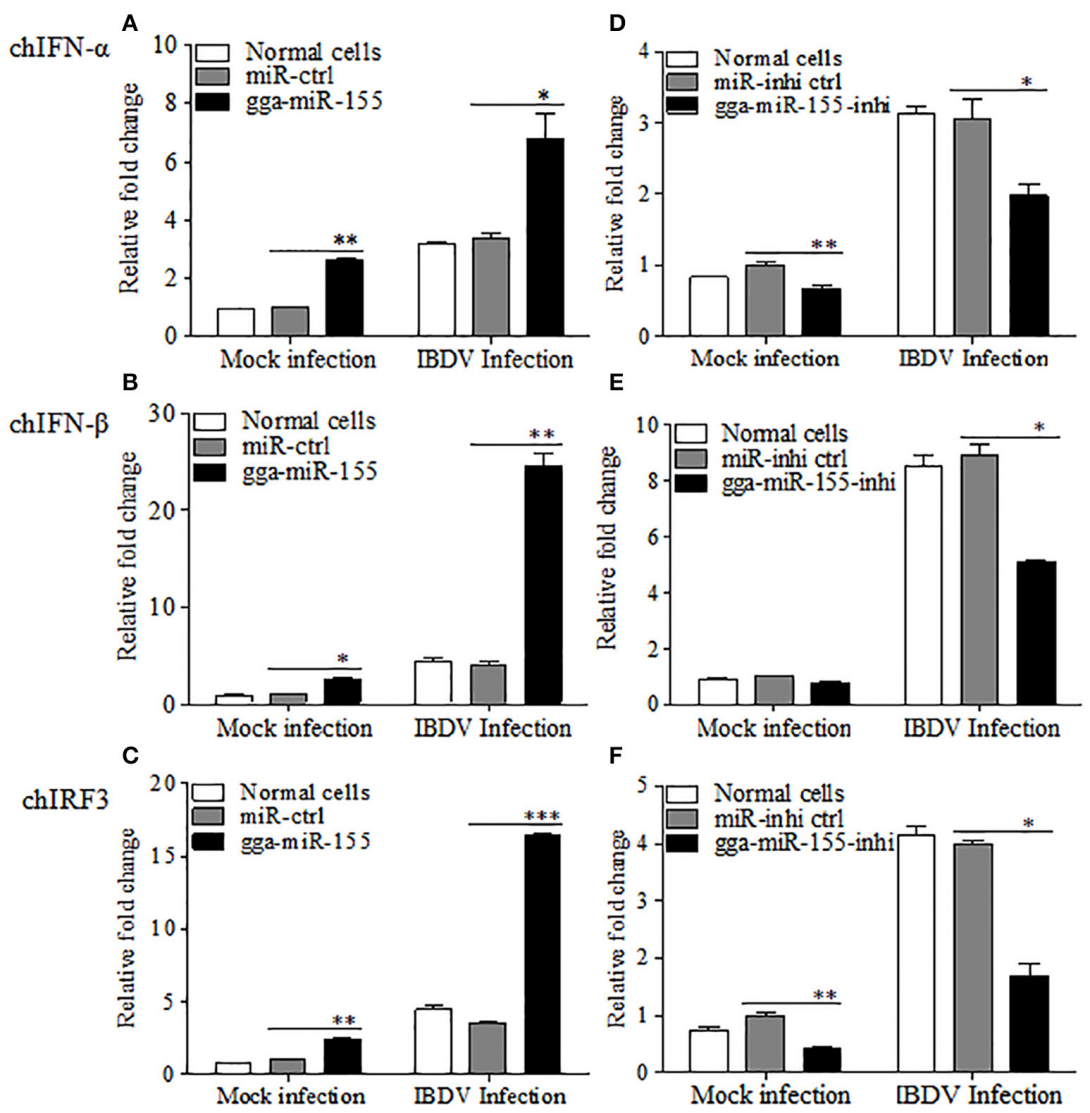
gga-miR-155 enhances IBDV-induced expressions of type I interferon and chIRF3 in DF-1 cells. **(A–C)** Transfection of gga-miR-155 enhances IBDV-induced expressions of type I interferon and chIRF3 in DF-1 cells. **(A–C)** DF-1 cells were transfected with gga-miR-155 mimics or miRNA mimic controls at the concentration of 200 nM. Twelve hours after tansfection, cells were infected with IBDV at an MOI of 0.01. Twenty-four hours after IBDV infection, cells were harvested for quantifying the expressions of chIFN-α **(A)**, chIFN-β **(B)** and chIRF3 **(C)** by qRT-PCR. The relative levels of chIFN-α, chIFN-β or chIRF3 mRNA expression is calculated as follows: the mRNA expressions of chIFN-α, chIFN-β or chIRF3 in gga-miR-155 transfected or normal cells infected with IBDV / miRNA control expressions in these transfected cells in mock control. GAPDH was used as an internal control. **(D–F)** Inhibition of endogenous gga-miR-155 by inhibitors reduced IBDV-induced expressions of type I interferon and chIRF3 in DF-1 cells. DF-1 cells were transfected with gga-miR-155-inhibitor mimics or miRNA-inhibitor control at the concentration of 200 nM. Twelve hours after tansfection, cells were infected with IBDV at an MOI of 0.01. Twenty-four hours after IBDV infection, cells were harvested for quantifying the expressions of chIFN-α **(D)**, chIFN-β **(E)**, and chIRF3 **(F)** by qRT-PCR. The relative levels of chIFN-α, chIFN-β or chIRF3 mRNA expression is calculated as follows: the mRNA expression of chIFN-α, chIFN-β or chIRF3 in gga-miR-155 inhibitor treated cells or normal cells infected with IBDV / that of miRNA inhibitor control treated cells in mock control. GAPDH was used as an internal control. Data are representative of three independent experiments and presented as mean ± SD. ^***^p < 0.001, ^**^p < 0.01 and ^*^p < 0.05.

As type I interferon plays a critical role in the host response against IBDV infection (O'Neill et al., 2010; Li et al., 2013), we hypothesized that gga-miR-155 inhibits IBDV replication by promoting type I interferon signaling. To test this hypothesis, we transfected DF-1 cells with gga-miR-155 and examined the expression of type I interferon in DF-1 cells after infection with IBDV at an MOI of 0.01 using qRT-PCR assay. We found that overexpression of gga-miR-155 significantly enhanced IBDV-induced expressions of chIFN-β and chIRF3 but not so much for chIFN-α in DF-1 cells, and the increase was about 5 and 3 folds for chIFN-β and chIRF3, respectively (Figures 4A–C). Furthermore, knockdown of endogenous gga-miR-155 by inhibitor markedly suppressed IBDV-induced expressions of chIFN-β and chIRF3 in DF-1 cells, reducing chicken type-I interferon and chIRF3 expressions about 2 folds (Figures 4D–F). These data suggest that gga-miR-155 enhances chicken type-I interferon and chIRF3 expressions in cell response to dsRNA virus infection.

### gga-miR-155 directly targets SOCS1 and TANK

**Figure 5:**
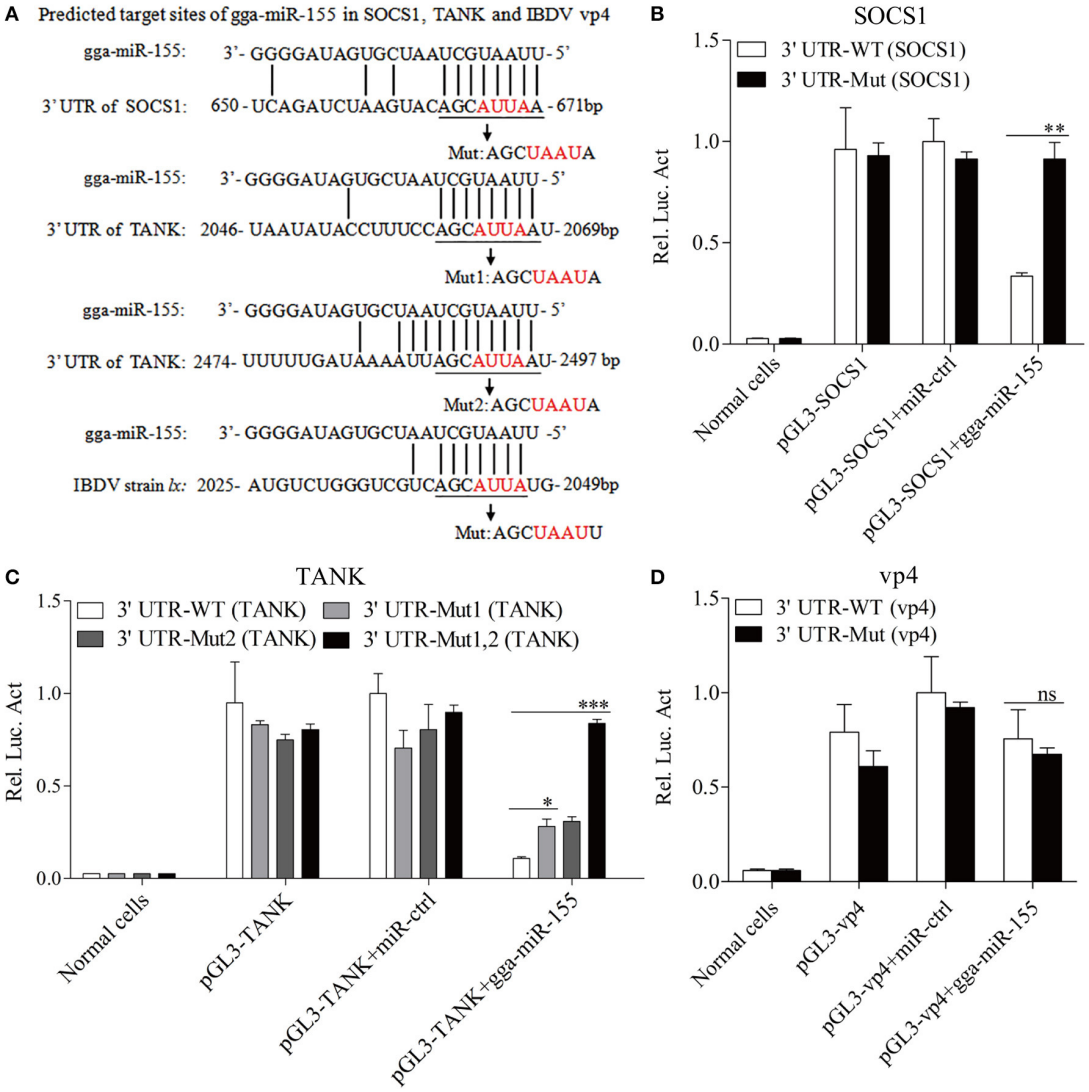
gga-miR-155 directly targets SOCS1 and TANK. **(A)** Diagram of the predicted target sites for gga-miR-155 in SOCS1, TANK and IBDV vp4. Seed regions of gga-miR-155 were underlined and mutated as indicated by the arrows. **(B,C)** Transfection of gga-miR-155 reduced expressions of SOCS1 and TANK but not their mutants. DF-1 cells were cotransfected with gga-miR-155 and WT or mutant SOCS1 or TANK luciferase reporter vectors. Thirty-six hours post-transfection, cells were lysed and luciferase reporter gene assays were performed to measure SOCS1 and TANK expressions. **(D)** gga-miR-155 did not target IBDV genomic RNA. DF-1 cells were transfected with gga-miR-155 and WT or mutant vp4 luciferase reporter vectors. Thirty-six hours post-transfection, cells were lysed and luciferase reporter gene assays were performed to measure vp4 expression. The relative level of luciferase activity is calculated as follows: luciferase activity of normal cells, reporter plasmid-transfected cells or cells transfected with the reporter plasmids and miRNA mimics/that of cells transfected with the wt reporter plasmids and miRNA control. Data are representative of three independent experiments and presented as means ± SD. ^***^p < 0.001, ^**^p < 0.01, and ^*^p < 0.05.

**Figure 6:**
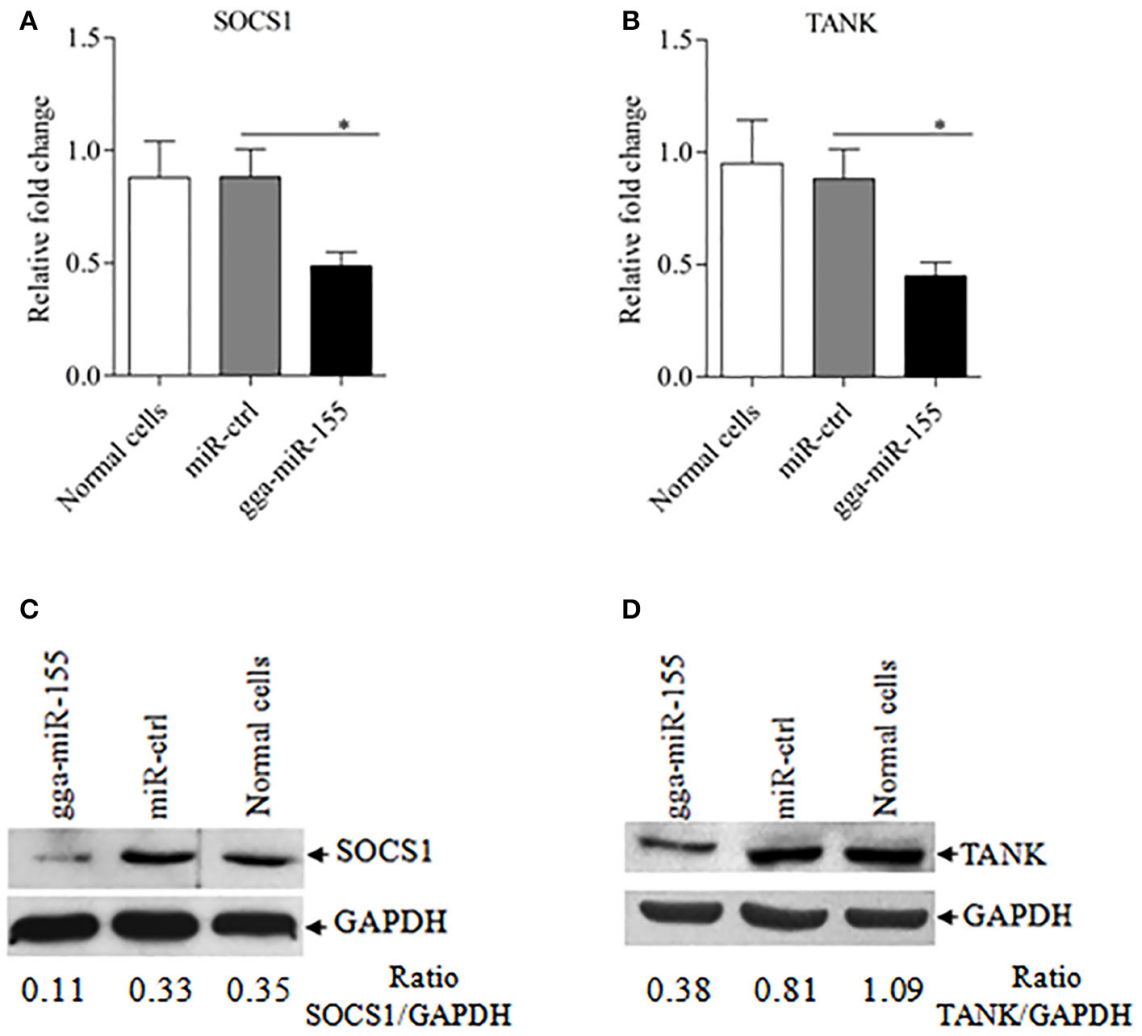
gga-miR-155 inhibits expressions of SOCS1 and TANK. **(A,B)** gga-miR-155 inhibits mRNA expressions of SOCS1 and TANK. DF-1 cells were transfected with gga-miR-155 mimics or miRNA mimic controls at the concentration of 200 nM. Twenty-four hours after transfection, mRNA expressions of SOCS1 **(A)** and TANK **(B)** were examined using qRT-PCR. The relative level of TANK mRNA expression is calculated as follows: the mRNA expression of SOCS1 or TANK in gga-miR-155 transfected cells or normal cells/that of miRNA treated control. GAPDH was used as an internal control. Data are representative of three independent experiments and presented as mean ± SD. ^*^p < 0.05. **(C,D)** Transfection of gga-miR-155 in DF-1 cells reduced expressions of SOCS1 and TANK at a protein level. DF-1 cells were transfected with gga-miR-155 mimics or miRNA mimic controls at the concentration of 200 nM. Thirty-six hours after transfection, cells were harvested and the cytosolic proteins were prepared and subjected to Western Blot assays for SOCS1 and TANK expressions **(C,D)**, and GAPDH was used as an internal control.

Using TargetScan and miRanda prediction softwares, we found two putative gga-miR-155 targeted genes *SOCS1* and *TANK* that likely modulate type I IFN signaling in DF-1 cells. Our result verified previous studies where SOCS1 was identified as a gga-mIR-155 target (Figueroa et al., 2016). It is well-known that SOCS1 and TANK are two negative regulators in immune response (Dai et al., 2006; Kawagoe et al., 2009; Fujimoto and Naka, 2010; Huang et al., 2015). Thus, it is logical to examine the effect of gga-miR-155 on the expressions of these two potential target molecules. To examine whether gga-miR-155 directly target seed regions in *SOCS1* and *TANK* as predicted, we constructed firefly luciferase reporter pGL3-3′UTR-WT (*SOCS1*) and pGL3-3′UTR-WT (*TANK*) containing the predicted target site in the 3′UTR, and four mutant vectors pGL3-3′UTR-Mut (*SOCS1*), pGL3-3′UTR-Mut1 (*TANK*), pGL3-3′UTR-Mut2 (*TANK*) and pGL3-3′UTR-Mut1,2 (*TANK*) with mutations of four nucleotides in the seed region (Figure 5A), and transfected DF-1 cells with these reporter gene plasmids and miRNA mimics. We found that gga-miR-155 significantly inhibited the luciferase activities of pGL3-3′UTR-WT (*SOCS1*) and pGL3-3′UTR-WT (*TANK*), but not pGL3-3′UTR-Mut (*SOCS1*) or pGL3-3′UTR-Mut (*TANK*) (Figures 5B,C). To rule out the possibility that gga-miR-155 might have potential target sites in IBDV genomic RNA, we examined the IBDV genomic RNA sequence and found a predicted target in IBDV *Lx* strain *vp4* gene using targetScan and miRanda. However, gga-miR-155 didn't have any effect on luciferase activity of pGL3-3′UTR-WT (*vp4*) (Figure 5D). Furthermore, overexpression of gga-miR-155 reduced the expressions of SOCS1 and TANK by around 2 folds in cells at both mRNA and protein levels (Figures 6A–D). It was previously found that gga-miR-155 targets SOCS1 (Figueroa et al., 2016), and our data not only confirmed this finding but also indicated the involvement of gga-miR-155 in targeting another host protein TANK. Thus, both SOCS1 and TANK are targeted and regulated by gga-miR-155.

### Knockdown of TANK enhanced Poly(I:C)-induced expressions of type I interferon and suppressed IBDV replication in DF-1 cells

**Figure 7:**
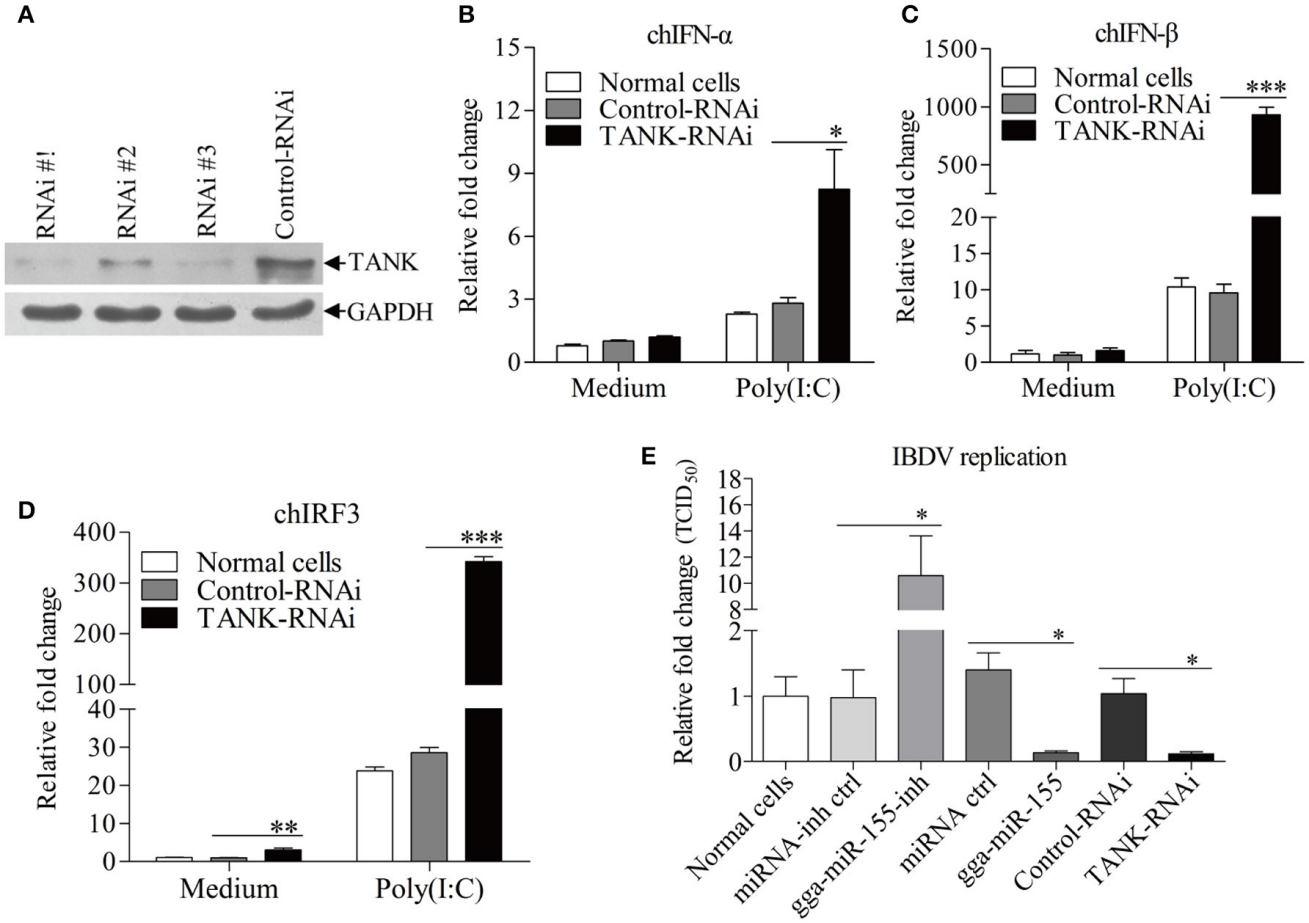
Knockdown of TANK enhanced poly(I:C)-induced expressions of chIFN-α, chIFN-β, and chIRF3 in DF-1 cells and suppresses IBDV replication. **(A)** Effects of TANK RNAi on the expression of endogenous TANK in DF-1 cells. DF-1 cells were transfected with siRNA constructs (RNAi #1 to #3) or controls. Double transfections were performed at a 24 h interval. Twenty-four hours after the second transfection, cell lysates were prepared and examined by Western Blot **(A)**, GAPDH was used as an internal control. **(B–D)** Knockdown of TANK enhanced poly(I:C)-induced expression of chIFN-α, chIFN-β, and chIRF3. DF-1 cells were transfected with RNAi constructs, twenty-four hours after the second transfection, cells were treated with poly (I: C) at a final concentration of 2 μg/ml for 12 h, followed by measurement of mRNA expressions of chIFN-α **(B)**, chIFN-β **(C)**, and chIRF3 **(D)** via qRT-PCR using specific primers. The relative level of chIFN-α, chIFN-β, or chIRF3 mRNA expression is calculated as follows: the mRNA expression of chIFN-α, chIFN-β, or chIRF3 in TANK-RNAi treated cells or normal cells/ that of control-RNAi treated cells in medium control. GAPDH was used as an internal control. **(E)** Knockdown of TANK by siRNA suppressed IBDV replication. DF-1 cells were transfected with TANK siRNA, siRNA controls, gga-miR-155 mimics, miRNA controls, gga-miR-155 inhibitor, miRNA-inhibitor controls, respectively. Twelve hours post transfection, cells were infected with IBDV at an MOI of 0.01. Twenty-four hours post infection viral titers in the cell cultures were determined by TCID_50_ on DF-1 cells. Data are representative of three independent experiments and presented as means ± SD. ^***^p < 0.001, ^**^p < 0.01, and ^*^p < 0.05.

To examine the role of TANK in suppression of viral replication by gga-miR-155, we made three TANK RNAi constructs, and we found that one could effectively lower the cellular level of TANK without causing discernible changes in cell morphology (Figure 7A). We then examined the expressions of type I interferon and chIRF3 in these cells receiving this siRNA or control siRNA after stimulation with poly(I:C). As a result, knockdown of TANK by RNAi markedly enhanced poly(I:C)-induced expression of type I interferon (Figures 7B,C) and chIRF3 (Figure 7D) in DF-1 cells. In contrast, IBDV replication was significantly suppressed by knockdown of TANK, similar to the effect of gga-miR-155 on IBDV replication, reducing the virus titers by around 10 folds as compared to that of the miRNA control (Figure 7E). As gga-miR-155 reduced the expression of TANK, these results indicate that gga-miR-155 enhances type I interferon and suppresses IBDV replication via reduction of TANK.

**Figure 8:**
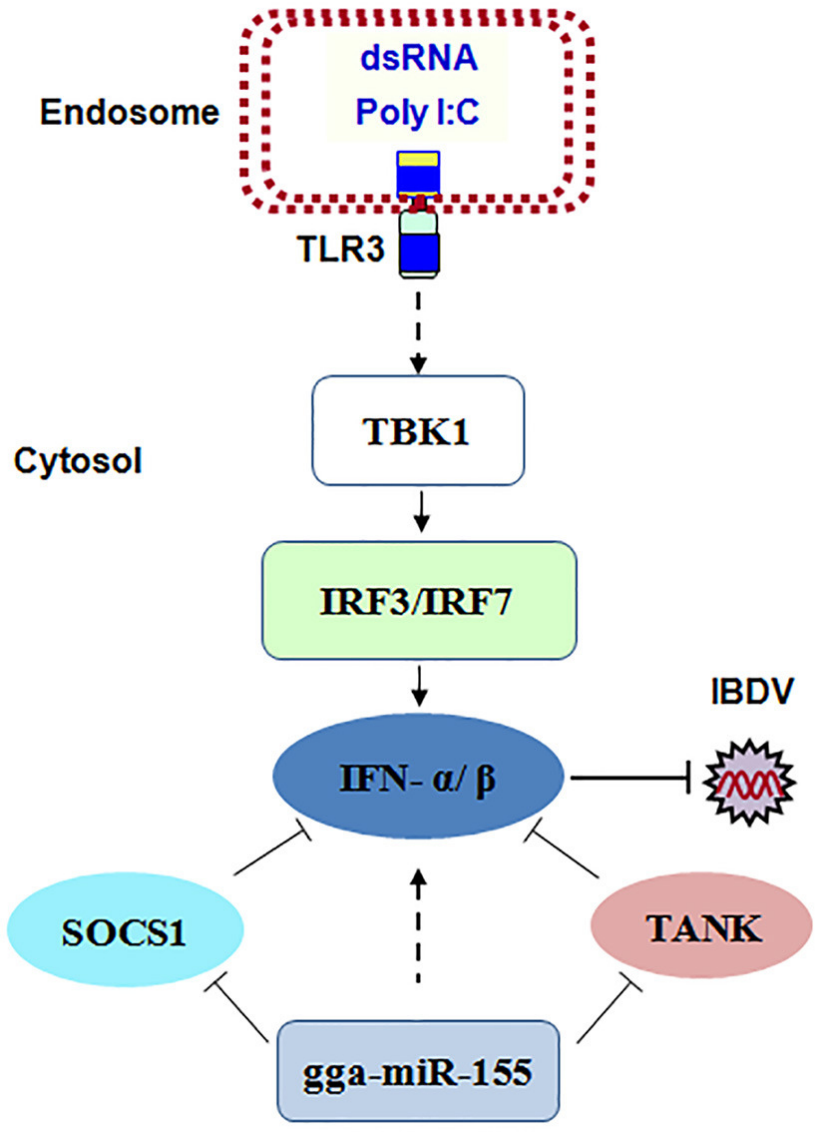
The model for gga-miR-155-mediated enhancement of IFN-α/β expression for the suppression of IBDV replication. Infection with IBDV, a double-stranded RNA virus, triggers TLR3 signaling pathway and gga-miR-155 expression. gga-miR-155 enhances IFN-α/β expression and suppresses infectious burse disease virus replication via targeting the negative regulators, SOCS1 and TANK.

Taken together, our data show that IBDV infection induces gga-miR-155 expression and that miR-155 enhances type I IFN signaling and suppresses IBDV replication by targeting SOCS1 and TANK, two negative regulators in the immune response (Figure 8).

## Discussion

IBDV infection causes severe damages in lymphoid organs in birds, especially the bursa of Fabricius (Müller et al., 2003). The surviving chickens with IBDV infection suffer from immunosuppression with compromised humoral and cellular immune responses (Sharma et al., 2000; van den Berg et al., 2000), leading to susceptibility of the chickens to other diseases. Although it was reported that several miRNAs are involved in regulating IBDV replication by targeting viral genomic RNA or host cell proteins (Wang et al., 2013; Ouyang et al., 2015), the effect of miRNAs on IBDV replication and the underlying mechanisms are still unclear. In this study, we evaluated the miRNA expression profile in DF-1 cells in response to IBDV infection and selected 11 candidate miRNAs for further analysis, and the 11 candidate miRNAs play important roles in host immunity (Gu et al., 2015; Hosokawa et al., 2015; Lamontagne et al., 2015; Lind et al., 2015; Tan et al., 2015; Wu et al., 2015; Gonzalez-Martin et al., 2016; Peng et al., 2016; Yan et al., 2016; Liang et al., 2017; Liu et al., 2017). Through TCID_50_ assay, we found that only gga-miR-155 significantly suppressed IBDV replication.

Mounting evidence suggest that miRNAs can coordinate host defense against viral infections (Thounaojam et al., 2014; Zhang et al., 2014; Li et al., 2015; Chen et al., 2016). Two mechanisms have been proposed (Wang et al., 2010, 2013). On one hand, multiple miRNAs can directly target viral genomes to inhibit virus replication (Song et al., 2010; Guo et al., 2013). On the other hand, miRNAs can trigger innate immune response or inhibit factors necessary for viral life cycle to enhance the host defense ability (Martinez-Nunez et al., 2009; Ren and Ambros, 2015). Similar to the second mechanism, gga-miR-155 inhibits IBDV replication by enhancing type I interferon signaling via targeting the negative regulators in innate immune system, SOCS1 and TANK. SOCS1 and TANK are important negative regulators in innate immune response (Dai et al., 2006; Kawagoe et al., 2009; Fujimoto and Naka, 2010; Huang et al., 2015). Our data show that overexpression of gga-miR-155 markedly reduced the expressions of SOCS1 and TANK in host cells and that knockdown of TANK by RNAi significantly enhanced poly(I:C)-induced expression of type I interferon and suppressed IBDV replication in cells. Clearly, gga-miR-155 enhances the type I interferon response of the host cells by targeting SOCS1 and TANK, leading to suppression of IBDV replication. As type I interferon is a critical to host defense against IBDV (Ragland et al., 2002; O'Neill et al., 2010), these results provide strong evidence that gga-miR-155, via targeting SOCS1 and TANK, enhances the innate immune response against IBDV infection. MiR-155 is transcribed from a noncoding gene named B-cell Integration Cluster (BIC) gene, which is highly conserved in many species such as human, mice, and chickens, and broadly expressed in various organs, tissues and cell types, indicating its versatile functions in various biological processes. As one of the best characterized miRNAs, miR-155 plays a critical role in various physiological and pathological processes such as immunity, inflammation, cancer, and viral infection (Wang et al., 2010; Pareek et al., 2014; Ji et al., 2016; Mantuano et al., 2016). Up to now, several genes have been reported as targets of miR-155, through which the biological function of miR-155 is elucidated, such as SHIP1, TGFβ-R2, SMAD5, and RUNX2 (O'Connell et al., 2009; Rai et al., 2010; Yin et al., 2010; George et al., 2015). Our data show that gga-miR-155 targets SOCS1 and TANK, enhancing type I interferon signaling in response to IBDV infection. However, it was reported that robust suppression of IFN-β occurred in the presence of miR-155 (Gracias et al., 2013; Hu et al., 2015). On the contrary, multiple lines of evidence have shown that miR-155 enhances type I interferon response of the host cells by targeting host protein, such as SOCS1 (Wang et al., 2010), SHIP1 (Thounaojam et al., 2014). In addition, miR-155 abrogates the suppressive effect of IL-10 and TGF-β on TLR3 signaling in HCV infection (Jiang et al., 2014). Furthermore, stimulation through TLR3 increases miR-155 levels in primary macrophages, diminishing HIV infection (Swaminathan et al., 2012). The discrepancy of these findings suggests that the suppression of IFN-β by gga-miR-155 might represent an alternative mechanism of gga-miR-155 action under a particular condition. It was possible that the low level of miR-155 expression enhanced type I interferon response while the high level of miR-155 would trigger a negative regulatory effect. Interestingly, miR-155-deficient CD8+ T cells did not produce IFN-α/β (Gracias et al., 2013). Meanwhile, DN-STAT1 and DN-IRF7 did not fully restore the response of miR-155-deficient OT-I cells, indicating that another signaling pathways may be affected by miR-155 deficiency (Gracias et al., 2013).

The interaction between hsa-miR-155 and SOCS1 has been reported in mammalian cells (Jiang et al., 2010), and gga-miR-155 has been previously shown to target SOCS1 (Figueroa et al., 2016). However, gga-miR-155's targeting chicken SOCS1 still needs to be testified, considering that similarity of amino acid sequences of chicken SOCS1 (GenBank accession no. NM001137648.1) with human SOCS1 (GenBank accession no. DQ086801.1) is only around 60.6%. Our data show that gga-miR-155 markedly suppressed SOCS1 expression. TANK plays versatile roles in innate immune signaling. On one hand, TANK serves as an adaptor bridging TRAF3 with TBK1 and IKKϵ, which promotes phosphorylation and activation of IRF3/IRF7 as well as induction of NF-κB activation, leading to efficient type I IFN production (Guo and Cheng, 2007; Ryzhakov and Randow, 2007). On the other hand, TANK has also been shown to act as a negative regulator of Toll-like receptor (TLRs) (Kawagoe et al., 2009; Wang et al., 2015). In this study, our data show that TANK serves as a negative regulator for type I interferon expression in chicken cells.

Of note, the mechanism underlying the interaction of host miRNAs and pathogenic infection is intricate. Host cells employ miRNAs to reduce pathogenic infection, while some pathogens encode miRNAs or manipulate host miRNAs expression to benefit their replication (Lu et al., 2008; Hu et al., 2015). Therefore, several questions are raised. For example, are there any other microRNAs than miR-155 in host cells that control IBDV replication? If so, what are they and what are their targets? Is avian miR-155 specific to IBDV infection? And why some pathogens can target miR-155 to suppress immune signaling to maintain their life cycle? (Lu et al., 2008; Hu et al., 2015) More efforts will be required to elucidate the molecular mechanisms underlying the pathogenesis of IBDV infection.

In summary, our results reveal that IBDV infection induces gga-miR-155 expression. We found that miR-155 enhanced type I IFN signaling and suppressed IBDV replication by targeted SOCS1 and TANK, two negative regulators in the immune response. These findings have provided insights for further studies of the molecular mechanism underlying host response to IBDV infection.

## Author contributions

SZ and BW designed and conceived the experiments, conducted data analysis and wrote the paper. BW and MF performed the experiments. YL, YW, XL, and HC contributed reagents, materials and analysis tools.

### Conflict of interest statement

The authors declare that the research was conducted in the absence of any commercial or financial relationships that could be construed as a potential conflict of interest. The reviewer AG and handling Editor declared their shared affiliation.

## References

[B1] Abdul-Careem M. F. Hunter B. D. Lee L. F. Fairbrother J. H. Haghighi H. R. Read L.. (2008). Host responses in the bursa of Fabricius of chickens infected with virulent Marek's disease virus. Virology 379, 256–265. 10.1016/j.virol.2008.06.027 18675437

[B2] Azad A. A. Barrett S. A. Fahey K. J. (1985). The characterization and molecular cloning of the double-stranded RNA genome of an Australian strain of infectious bursal disease virus. Virology 143, 35–44. 2998012 10.1016/0042-6822(85)90094-7

[B3] Baltimore D. Boldin M. P. O'Connell R. M. Rao D. S. Taganov K. D. (2008). MicroRNAs: new regulators of immune cell development and function. Nat. Immunol. 9, 839–845. 10.1038/ni.f.209 18645592

[B4] Cai M. Zhu F. Shen P. (2012). Expression and purification of chicken beta interferon and its antivirus immunological activity. Protein Expr. Purif. 84, 123–129. 10.1016/j.pep.2012.04.014 22564876

[B5] Chen L. Song Y. He L. Wan X. Lai L. Dai F.. (2016). MicroRNA-223 promotes type I interferon production in antiviral innate immunity by targeting Forkhead Box Protein O3 (FOXO3). J. Biol. Chem. 291, 14706–14716. 10.1074/jbc.M115.700252 27226534 PMC4938189

[B6] Dai X. Sayama K. Yamasaki K. Tohyama M. Shirakata Y. Hanakawa Y.. (2006). SOCS1-negative feedback of STAT1 activation is a key pathway in the dsRNA-induced innate immune response of human keratinocytes. J. Invest. Dermatol. 126, 1574–1581. 10.1038/sj.jid.5700294 16628196

[B7] Dai Z. Ji J. Yan Y. Lin W. Li H. Chen F.. (2015). Role of gga-miR-221 and gga-miR-222 during Tumour Formation in Chickens Infected by Subgroup J Avian Leukosis Virus. Viruses 7, 6538–6551. 10.3390/v7122956 26690468 PMC4690879

[B7a] Figueroa T. Boumart I. Coupeau D. Rasschaert D. (2016). Hyperediting by ADAR1 of a new herpesvirus lncRNA during the lytic phase of the oncogenic Marek's disease virus. J. Gen. Virol. 97, 2973–2988. 10.1099/jgv.0.000606 27655063

[B8] Fujimoto M. Naka T. (2010). SOCS1, a Negative Regulator of Cytokine Signals and TLR Responses, in Human Liver Diseases. Gastroenterol Res Pract 2010:470468. 10.1155/2010/470468 20862390 PMC2939392

[B9] George J. Lewis M. G. Renne R. Mattapallil J. J. (2015). Suppression of transforming growth factor beta receptor 2 and Smad5 is associated with high levels of microRNA miR-155 in the oral mucosa during chronic simian immunodeficiency virus infection. J. Virol. 89, 2972–2978. 10.1128/JVI.03248-14 25540365 PMC4325739

[B10] Gonzalez-Martin A. Adams B. D. Lai M. Shepherd J. Salvador-Bernaldez M. Salvador J. M.. (2016). The microRNA miR-148a functions as a critical regulator of B cell tolerance and autoimmunity. Nat. Immunol. 17, 433–440. 10.1038/ni.3385 26901150 PMC4803625

[B11] Gracias D. T. Stelekati E. Hope J. L. Boesteanu A. C. Doering T. A. Norton J.. (2013). The microRNA miR-155 controls CD8(+) T cell responses by regulating interferon signaling. Nat. Immunol. 14, 593–602. 10.1038/ni.2576 23603793 PMC3664306

[B12] Gu C. Zhou X. D. Yuan Y. Miao X. H. Liu Y. Ru Y. W.. (2015). MicroRNA-214 induces dendritic cell switching from tolerance to immunity by targeting beta-Catenin signaling. Int. J. Clin. Exp. Pathol. 8, 10050–10060. 26617712 PMC4637527

[B13] Guo B. Cheng G. (2007). Modulation of the interferon antiviral response by the TBK1/IKKi adaptor protein TANK. J. Biol. Chem. 282, 11817–11826. 10.1074/jbc.M700017200 17327220

[B14] Guo X. K. Zhang Q. Gao L. Li N. Chen X. X. Feng W. H. (2013). Increasing expression of microRNA 181 inhibits porcine reproductive and respiratory syndrome virus replication and has implications for controlling virus infection. J. Virol. 87, 1159–1171. 10.1128/JVI.02386-12 23152505 PMC3554091

[B15] He L. Hannon G. J. (2004). MicroRNAs: small RNAs with a big role in gene regulation. Nat. Rev. Genet. 5, 522–531. 10.1038/nrg1379 15211354

[B16] Hosokawa K. Muranski P. Feng X. Keyvanfar K. Townsley D. M. Dumitriu B.. (2015). Identification of novel microRNA signatures linked to acquired aplastic anemia. Haematologica 100, 1534–1545. 10.3324/haematol.2015.126128 26354756 PMC4666329

[B17] Hu X. Ye J. Qin A. Zou H. Shao H. Qian K. (2015). Both MicroRNA-155 and Virus-Encoded MiR-155 Ortholog Regulate TLR3 Expression. PLoS ONE 10:e0126012. 10.1371/journal.pone.0126012 25938551 PMC4418834

[B18] Huang L. Liu Q. Zhang L. Zhang Q. Hu L. Li C.. (2015). Encephalomyocarditis Virus 3C Protease Relieves TRAF Family Member-associated NF-kappaB Activator (TANK) Inhibitory Effect on TRAF6-mediated NF-kappaB Signaling through Cleavage of TANK. J. Biol. Chem. 290, 27618–27632. 10.1074/jbc.M115.660761 26363073 PMC4646013

[B19] Hudson P. J. McKern N. M. Power B. E. Azad A. A. (1986). Genomic structure of the large RNA segment of infectious bursal disease virus. Nucleic Acids Res. 14, 5001-5012. 3014441 10.1093/nar/14.12.5001 PMC311506

[B20] Jagadish M. N. Staton V. J. Hudson P. J. Azad A. A. (1988). Birnavirus precursor polyprotein is processed in Escherichia coli by its own virus-encoded polypeptide. J. Virol. 62, 1084–1087. 2828658 10.1128/jvi.62.3.1084-1087.1988 PMC253673

[B21] Ji J. Xu M. Tu J. Zhao Z. Gao J. Chen M.. (2016). MiR-155 and its functional variant rs767649 contribute to the susceptibility and survival of Hepatocellular carcinoma. 7, 60303–60309. 10.18632/oncotarget.11206 27531892 PMC5312385

[B22] Jiang M. Broering R. Trippler M. Wu J. Zhang E. Zhang X.. (2014). MicroRNA-155 controls Toll-like receptor 3- and hepatitis C virus-induced immune responses in the liver. J. Viral Hepat. 21, 99–110. 10.1111/jvh.12126 24383923

[B23] Jiang S. Zhang H. W. Lu M. H. He X. H. Li Y. Gu H.. (2010). MicroRNA-155 functions as an OncomiR in breast cancer by targeting the suppressor of cytokine signaling 1 gene. Cancer Res. 70, 3119–3127. 10.1158/0008-5472.CAN-09-4250 20354188

[B24] Kawagoe T. Takeuchi O. Takabatake Y. Kato H. Isaka Y. Tsujimura T.. (2009). TANK is a negative regulator of Toll-like receptor signaling and is critical for the prevention of autoimmune nephritis. Nat. Immunol. 10, 965–972. 10.1038/ni.1771 19668221 PMC2910115

[B25] Kibenge F. S. McKenna P. K. Dybing J. K. (1991). Genome cloning and analysis of the large RNA segment (segment A) of a naturally avirulent serotype 2 infectious bursal disease virus. Virology 184, 437–440. 1651602 10.1016/0042-6822(91)90865-9

[B26] Lamontagne J. Steel L. F. Bouchard M. J. (2015). Hepatitis B virus and microRNAs: complex interactions affecting hepatitis B virus replication and hepatitis B virus-associated diseases. World J. Gastroenterol. 21, 7375–7399. 10.3748/wjg.v21.i24.7375 26139985 PMC4481434

[B27] Lewis B. P. Burge C. B. Bartel D. P. (2005). Conserved seed pairing, often flanked by adenosines, indicates that thousands of human genes are microRNA targets. Cell 120, 15–20. 10.1016/j.cell.2004.12.035 15652477

[B28] Li L. Wei Z. Zhou Y. Gao F. Jiang Y. Yu L.. (2015). Host miR-26a suppresses replication of porcine reproductive and respiratory syndrome virus by upregulating type I interferons. Virus Res. 195, 86–94. 10.1016/j.virusres.2014.08.012 25218480 PMC7114497

[B29] Li Y. P. Handberg K. J. Juul-Madsen H. R. Zhang M. F. Jorgensen P. H. (2007). Transcriptional profiles of chicken embryo cell cultures following infection with infectious bursal disease virus. Arch. Virol. 152, 463–478. 10.1007/s00705-006-0878-9 17143781

[B30] Li Z. Wang Y. Li X. Li X. Cao H. Zheng S. J. (2013). Critical roles of glucocorticoid-induced leucine zipper in infectious bursal disease virus (IBDV)-induced suppression of type I Interferon expression and enhancement of IBDV growth in host cells via interaction with VP4. J. Virol. 87, 1221–1231. 10.1128/JVI.02421-12 23152515 PMC3554076

[B31] Liang X. Shangguan W. Zhang M. Mei S. Wang L. Yang R. (2017). miR-128 enhances dendritic cell-mediated anti-tumor immunity via targeting of p38. Mol. Med. Rep. 16, 1307–1313. 10.3892/mmr.2017.6717 29067466 PMC5561786

[B32] Lind E. F. Millar D. G. Dissanayake D. Savage J. C. Grimshaw N. K. Kerr W. G.. (2015). miR-155 upregulation in dendritic cells is sufficient to break tolerance *in vivo* by negatively regulating SHIP1. J. Immunol. 195, 4632–4640. 10.4049/jimmunol.1302941 26447227

[B33] Liu F. Liu C. Hu X. Shang Y. Wu L. (2017). MicroRNA-21: a positive regulator for optimal production of Type I and Type III interferon by plasmacytoid dendritic cells. Front Immunol 8:947. 10.3389/fimmu.2017.00947 28871250 PMC5567078

[B34] Liu H. Zhang M. Han H. Yuan J. Li Z. (2010). Comparison of the expression of cytokine genes in the bursal tissues of the chickens following challenge with infectious bursal disease viruses of varying virulence. Virol. J. 7:364. 10.1186/1743-422X-7-364 21143846 PMC3004833

[B35] Lu F. Weidmer A. Liu C. G. Volinia S. Croce C. M. Lieberman P. M. (2008). Epstein-Barr virus-induced miR-155 attenuates NF-kappaB signaling and stabilizes latent virus persistence. J. Virol. 82, 10436–10443. 10.1128/JVI.00752-08 18753206 PMC2573162

[B36] Mantuano E. Brifault C. Lam M. S. Azmoon P. Gilder A. S. Gonias S. L. (2016). LDL receptor-related protein-1 regulates NFkappaB and microRNA-155 in macrophages to control the inflammatory response. Proc. Natl. Acad. Sci. U.S.A. 113, 1369–1374. 10.1073/pnas.1515480113 26787872 PMC4747752

[B37] Martinez-Nunez R. T. Louafi F. Friedmann P. S. Sanchez-Elsner T. (2009). MicroRNA-155 modulates the pathogen binding ability of dendritic cells (DCs) by down-regulation of DC-specific intercellular adhesion molecule-3 grabbing non-integrin (DC-SIGN). J. Biol. Chem. 284, 16334–16342. 10.1074/jbc.M109.011601 19386588 PMC2713543

[B38] Morgan M. M. Macreadie I. G. Harley V. R. Hudson P. J. Azad A. A. (1988). Sequence of the small double-stranded RNA genomic segment of infectious bursal disease virus and its deduced 90-kDa product. Virology 163, 240–242. 2831661 10.1016/0042-6822(88)90258-9

[B39] Müller H. Islam M. R. Raue R. (2003). Research on infectious bursal disease–the past, the present and the future. Vet. Microbiol. 97, 153–165. 10.1016/j.vetmic.2003.08.005 14637046

[B40] O'Connell R. M. Chaudhuri A. A. Rao D. S. Baltimore D. (2009). Inositol phosphatase SHIP1 is a primary target of miR-155. Proc. Natl. Acad. Sci. U.S.A. 106, 7113–7118. 10.1073/pnas.0902636106 19359473 PMC2678424

[B41] O'Connell R. M. Rao D. S. Baltimore D. (2012). microRNA regulation of inflammatory responses. Annu. Rev. Immunol. 30, 295–312. 10.1146/annurev-immunol-020711-075013 22224773

[B42] O'Connell R. M. Taganov K. D. Boldin M. P. Cheng G. Baltimore D. (2007). MicroRNA-155 is induced during the macrophage inflammatory response. Proc. Natl. Acad. Sci. U.S.A. 104, 1604–1609. 10.1073/pnas.0610731104 17242365 PMC1780072

[B43] O'Neill A. M. Livant E. J. Ewald S. J. (2010). Interferon alpha-induced inhibition of infectious bursal disease virus in chicken embryo fibroblast cultures differing in Mx genotype. Avian Dis. 54, 802–806. 10.1637/9001-072309-Reg.1 20608522

[B44] Ouyang W. Wang Y. S. Du X. N. Liu H. J. Zhang H. B. (2015). gga-miR-9^*^ inhibits IFN production in antiviral innate immunity by targeting interferon regulatory factor 2 to promote IBDV replication. Vet. Microbiol. 178, 41–49. 10.1016/j.vetmic.2015.04.023 25975521

[B45] Pan J. Lin L. Tao Y. J. (2009). Self-guanylylation of birnavirus VP1 does not require an intact polymerase activity site. Virology 395, 87–96. 10.1016/j.virol.2009.09.004 19801157 PMC2783171

[B46] Pareek S. Roy S. Kumari B. Jain P. Banerjee A. Vrati S. (2014). MiR-155 induction in microglial cells suppresses Japanese encephalitis virus replication and negatively modulates innate immune responses. J. Neuroinflammation 11:97. 10.1186/1742-2094-11-97 24885259 PMC4050406

[B47] Peng C. Wang H. Zhang W. J. Jie S. H. Tong Q. X. Lu M. J.. (2016). Inhibitory effect of miR-125b on hepatitis C virus core protein-induced TLR2/MyD88 signaling in THP-1 cells. World J. Gastroenterol. 22, 4354–4361. 10.3748/wjg.v22.i17.4354 27158204 PMC4853693

[B48] Pitcovski J. Gutter B. Gallili G. Goldway M. Perelman B. Gross G.. (2003). Development and large-scale use of recombinant VP2 vaccine for the prevention of infectious bursal disease of chickens. Vaccine 21, 4736–4743. 10.1016/S0264-410X(03)00525-5 14585684

[B49] Ragland W. L. Novak R. El-Attrache J. Savic V. Ester K. (2002). Chicken anemia virus and infectious bursal disease virus interfere with transcription of chicken IFN-alpha and IFN-gamma mRNA. J. Interferon. Cytokine Res. 22, 437–441. 10.1089/10799900252952226 12034026

[B50] Rai D. Kim S. W. McKeller M. R. Dahia P. L. Aguiar R. C. (2010). Targeting of SMAD5 links microRNA-155 to the TGF-beta pathway and lymphomagenesis. Proc. Natl. Acad. Sci. U.S.A. 107, 3111–3116. 10.1073/pnas.0910667107 20133617 PMC2840369

[B51] Reed L. J. Muench M. H. (1938). A simple method of estimating fifty percent endpoints. Am. J. Epidemiol. 27, 493–497.

[B52] Ren Z. Ambros V. R. (2015). Caenorhabditis elegans microRNAs of the let-7 family act in innate immune response circuits and confer robust developmental timing against pathogen stress. Proc. Natl. Acad. Sci. U.S.A. 112, E2366–E2375. 10.1073/pnas.1422858112 25897023 PMC4426397

[B53] Ryzhakov G. Randow F. (2007). SINTBAD, a novel component of innate antiviral immunity, shares a TBK1-binding domain with NAP1 and TANK. EMBO J. 26, 3180–3190. 10.1038/sj.emboj.7601743 17568778 PMC1914091

[B54] Sharma J. M. Kim I. J. Rautenschlein S. Yeh H. Y. (2000). Infectious bursal disease virus of chickens: pathogenesis and immunosuppression. Dev. Comp. Immunol. 24, 223–235. 10.1016/S0145-305X(99)00074-9 10717289

[B55] Song L. Liu H. Gao S. Jiang W. Huang W. (2010). Cellular microRNAs inhibit replication of the H1N1 influenza A virus in infected cells. J. Virol. 84, 8849–8860. 10.1128/JVI.00456-10 20554777 PMC2919005

[B56] Stricker R. L. Behrens S. E. Mundt E. (2010). Nuclear factor NF45 interacts with viral proteins of infectious bursal disease virus and inhibits viral replication. J. Virol. 84, 10592–10605. 10.1128/JVI.02506-09 20702628 PMC2950606

[B57] Swaminathan G. Rossi F. Sierra L. J. Gupta A. Navas-Martín S. Martín-García J. (2012). A role for microRNA-155 modulation in the anti-HIV-1 effects of Toll-like receptor 3 stimulation in macrophages. PLoS Pathog 8:e1002937. 10.1371/journal.ppat.1002937 23028330 PMC3447756

[B58] Tacken M. G. Thomas A. A. Peeters B. P. Rottier P. J. Boot H. J. (2004). VP1, the RNA-dependent RNA polymerase and genome-linked protein of infectious bursal disease virus, interacts with the carboxy-terminal domain of translational eukaryotic initiation factor 4AII. Arch. Virol. 149, 2245–2260. 10.1007/s00705-004-0365-0 15503210

[B59] Tan Y. Lin B. Ye Y. Wen D. Chen L. Zhou X. (2015). Differential expression of serum microRNAs in cirrhosis that evolve into hepatocellular carcinoma related to hepatitis B virus. Oncol. Rep. 33, 2863–2870. 10.3892/or.2015.3924 25962820

[B60] Thounaojam M. C. Kundu K. Kaushik D. K. Swaroop S. Mahadevan A. Shankar S. K.. (2014). MicroRNA 155 regulates Japanese encephalitis virus-induced inflammatory response by targeting Src homology 2-containing inositol phosphatase 1. J. Virol. 88, 4798–4810. 10.1128/JVI.02979-13 24522920 PMC3993824

[B61] Tong L. Lin L. Wu S. Guo Z. Wang T. Qin Y.. (2013). MiR-10a^*^ up-regulates coxsackievirus B3 biosynthesis by targeting the 3D-coding sequence. Nucleic Acids Res. 41, 3760–3771. 10.1093/nar/gkt058 23389951 PMC3616696

[B62] van den Berg T. P. Eterradossi N. Toquin D. Meulemans G. (2000). Infectious bursal disease (Gumboro disease). Rev. Sci. Tech. 19, 509–543. 10935278

[B63] Wang P. Hou J. Lin L. Wang C. Liu X. Li D.. (2010). Inducible microRNA-155 feedback promotes type I IFN signaling in antiviral innate immunity by targeting suppressor of cytokine signaling 1. J. Immunol. 185, 6226–6233. 10.4049/jimmunol.1000491 20937844

[B64] Wang W. Huang X. Xin H. B. Fu M. Xue A. Wu Z. H. (2015). TRAF Family Member-associated NF-kappaB Activator (TANK) Inhibits Genotoxic Nuclear Factor kappaB Activation by Facilitating Deubiquitinase USP10-dependent Deubiquitination of TRAF6 Ligase. J. Biol. Chem. 290, 13372–13385. 10.1074/jbc.M115.643767 25861989 PMC4505586

[B65] Wang Y. S. Ouyang W. Pan Q. X. Wang X. L. Xia X. X. Bi Z. W.. (2013). Overexpression of microRNA gga-miR-21 in chicken fibroblasts suppresses replication of infectious bursal disease virus through inhibiting VP1 translation. Antiviral Res. 100, 196–201. 10.1016/j.antiviral.2013.08.001 23954191

[B66] Wu W. He C. Liu C. Cao A. T. Xue X. Evans-Marin H. L.. (2015). miR-10a inhibits dendritic cell activation and Th1/Th17 cell immune responses in IBD. Gut 64, 1755–1764. 10.1136/gutjnl-2014-307980 25281418

[B67] Yan H. Chen Y. Zhou S. Li C. Gong G. Chen X.. (2016). Expression Profile Analysis of miR-221 and miR-222 in Different Tissues and Head Kidney Cells of Cynoglossus semilaevis, Following Pathogen Infection. Mar. Biotechnol. 18, 37–48. 10.1007/s10126-015-9668-2 26420296

[B68] Yin Q. Wang X. Fewell C. Cameron J. Zhu H. Baddoo M.. (2010). MicroRNA miR-155 inhibits bone morphogenetic protein (BMP) signaling and BMP-mediated Epstein-Barr virus reactivation. J. Virol. 84, 6318–6327. 10.1128/JVI.00635-10 20427544 PMC2903268

[B69] Zhang J. Zhao H. Chen J. Xia B. Jin Y. Wei W.. (2012). Interferon-beta-induced miR-155 inhibits osteoclast differentiation by targeting SOCS1 and MITF. FEBS Lett. 586, 3255–3262. 10.1016/j.febslet.2012.06.047 22771905

[B70] Zhang Q. Guo X. K. Gao L. Huang C. Li N. Jia X. (2014). MicroRNA-23 inhibits PRRSV replication by directly targeting PRRSV RNA and possibly by upregulating type I interferons. Virology 450–451, 182–195. 10.1016/j.virol.2013.12.020 24503081

