# Corrigendum: gga-miR-155 Enhances Type I Interferon Expression and Suppresses Infectious Burse Disease Virus Replication via Targeting SOCS1 and TANK

**DOI:** 10.3389/fcimb.2020.00324

**Published:** 2020-07-15

**Authors:** Bin Wang, Mengjiao Fu, Yanan Liu, Yongqiang Wang, Xiaoqi Li, Hong Cao, Shijun J. Zheng

**Affiliations:** ^1^State Key Laboratory of Agrobiotechnology and College of Veterinary Medicine, China Agricultural University, Beijing, China; ^2^Key Laboratory of Animal Epidemiology of the Ministry of Agriculture, China Agricultural University, Beijing, China; ^3^Department of Preventive Veterinary Medicine, College of Veterinary Medicine, China Agricultural University, Beijing, China

**Keywords:** microRNA, type I IFN, IBDV, SOCS, TANK

In the original article, there was an error as Figueroa et al. 2016 was not cited. A correction has been made to the **Results** section, subsection **gga-miR-155 directly targets SOCS1 and TANK**:

“Using TargetScan and miRanda prediction softwares, we found two putative gga-miR-155 targeted genes *SOCS1* and *TANK* that likely modulate type I IFN signaling in DF-1 cells. Our result verified previous studies where SOCS1 was identified as a gga-mIR-155 target (Figueroa et al., 2016). It is well-known that SOCS1 and TANK are two negative regulators in immune response (Dai et al., 2006; Kawagoe et al., 2009; Fujimoto and Naka, 2010; Huang et al., 2015). Thus, it is logical to examine the effect of gga-miR-155 on the expressions of these two potential target molecules. To examine whether gga-miR-155 directly target seed regions in *SOCS1* and *TANK* as predicted, we constructed firefly luciferase reporter pGL3-3′UTR-WT (*SOCS1*) and pGL3-3′UTR-WT (*TANK*) containing the predicted target site in the 3′UTR, and four mutant vectors pGL3-3′UTR-Mut (*SOCS1*), pGL3-3′UTR-Mut1 (*TANK*), pGL3-3′UTR-Mut2 (*TANK*) and pGL3-3′UTR-Mut1,2 (*TANK*) with mutations of four nucleotides in the seed region (Figure 5A), and transfected DF-1 cells with these reporter gene plasmids and miRNA mimics. We found that gga-miR-155 significantly inhibited the luciferase activities of pGL3-3′UTR-WT (*SOCS1*) and pGL3-3′UTR-WT (*TANK*), but not pGL3-3′UTR-Mut (*SOCS1*) or pGL3-3′UTR-Mut (*TANK*) (Figures 5B,C). To rule out the possibility that gga-miR-155 might have potential target sites in IBDV genomic RNA, we examined the IBDV genomic RNA sequence and found a predicted target in IBDV *Lx* strain *vp4* gene using targetScan and miRanda. However, gga-miR-155 didn't have any effect on luciferase activity of pGL3-3′UTR-WT (*vp4*) (Figure 5D). Furthermore, overexpression of gga-miR-155 reduced the expressions of SOCS1 and TANK by around 2 folds in cells at both mRNA and protein levels (Figures 6A–D). It was previously found that gga-miR-155 targets SOCS1 (Figueroa et al., 2016), and our data not only confirmed this finding but also indicated the involvement of gga-miR-155 in targeting another host protein TANK. Thus, both SOCS1 and TANK are targeted and regulated by gga-miR-155.”

Secondly, the first sentence of the **Discussion** section, paragraph 3 was rephrased in order to clarify its meaning:

“The interaction between hsa-miR-155 and SOCS1 has been reported in mammalian cells (Jiang et al., 2010), and gga-miR-155 has been previously shown to target SOCS1 (Figueroa et al., 2016). However, gga-miR-155's targeting chicken SOCS1 still needs to be testified, considering that similarity of amino acid sequences of chicken SOCS1 (GenBank accession no. NM001137648.1) with human SOCS1 (GenBank accession no. DQ086801.1) is only around 60.6%. Our data show that gga-miR-155 markedly suppressed SOCS1 expression. TANK plays versatile roles in innate immune signaling. On one hand, TANK serves as an adaptor bridging TRAF3 with TBK1 and IKKϵ, which promotes phosphorylation and activation of IRF3/IRF7 as well as induction of NF-κB activation, leading to efficient type I IFN production (Guo and Cheng, 2007; Ryzhakov and Randow, 2007). On the other hand, TANK has also been shown to act as a negative regulator of Toll-like receptor (TLRs) (Kawagoe et al., 2009; Wang et al., 2015). In this study, our data show that TANK serves as a negative regulator for type I interferon expression in chicken cells.”

Also, the type of primer used for SOCS1 RT-qPCR was missing. The correct ones were synthesized by Sangon Biotech. A correction has been made to the **Materials and Methods** section, subsection **RNA isolation and quantitative real-time PCR (qRT-PCR) analysis**:

“Total RNA and miRNA were prepared from DF-1 cells using EASYspin Plus kit or RNA mini kit (aidlab Biotechnology, China) per the manufacturer's instructions. mRNAs were reversely transcribed with primescript^TM^ RT Reagent kit (Takara). Quantitative RT-PCR analysis was performed using Tli RnaseH Plus (Takara) on LightCycler 480II (Roche, USA). Specific primers for chicken IFN-α (chIFN-α) (5′-CCA GCA CCT CGA GCA AT-3′ and 5′-GGC GCT GTA ATC GTT GTC T-3′), chicken IFN-β (chIFN-β) (5′-GCC TCC AGC TCC TTC AGA ATA CG-3′ and 5′-CTG GAT CTG GTT GAG GAG GCT GT-3′), chicken IRF3 (chIF3) (5′-GCT CTC TGA CTC TTT CAA CCT CTT-3′ and 5′-AAT GCT GCT CTT TTC TCC TCT G-3′), and chicken GAPDH (5′-TGC CAT CAC AGC CAC ACA GAA G-3′ and 5′-ACT TTC CCC ACA GCC TTA GCA G-3′) were designed with reference to previous publications (Li et al., 2007; Abdul-Careem et al., 2008; Liu et al., 2010). Specific primers for chicken SOCS1 (5′- CTC GCA AGC GGA TTT CAG TAG-3′, 5′- GGG CTC AGA CTT CAG CTT CTC-3′) were designed and synthesized by Sangon Biotech (Shanghai, China). GAPDH gene was utilized as the reference gene. All quantitative real-time PCR experiments were performed in triplicate. The PCR was performed in a 20 μl volume containing 1 μl of cDNA, 10 μl of 2 × SYBR green Premix *Ex Taq* (TaKaRa), and a 0.4 μM of each gene-specific primers. Thermal cycling parameters were as follows: 94°C for 2 min; 40 cycles of 94°C for 20 s, 55°C for 20 s, and 72°C for 20 s; and 1 cycle of 95°C for 30 s, 60°C for 30 s, and 95°C for 30 s. The final step was to obtain a melt curve for the PCR products to determine the specificity of the amplification. qRT-PCR analysis of gga-miR-155 was performed with RT-PCR Quantitation Kit (GenePharma, China).”

Finally, the source of the SOCS1 antibody was also missing. A correction has been made to the **Materials and Methods** section, subsection **Western Blot analysis**:

“Chicken *socs1* gene was cloned from DF-1 cells using the specific primes 5′- CGC GGA TCC ATG GTA GCG CAC AGC AAG GTG-3′ (sense), and 5′- ACG CGT CGA CG TTA GAT CTG AAA CGG GAA GGA-3′ (anti-sense) with reference to the sequence in GenBank (accession no.NM_001137648.1). Chicken socs1 gene was then subcloned into pET-28a vector. The pET-28a-socs1 recombinant plasmid was used to transform *E. coli* BL21, and SOCS1-his recombinant protein was expressed at 37°C and purified by a protein purification kit (Ni Sepharose™ 6 Fast Flow, GE Company, USA) per the manufacturer's instructions. To develop polyclonal antibody against SOCS1, BALB/c mice were immunized with SOCS1-his recombinant protein. After immunization, anti-SOCS1 polyclonal antibody was obtained from the sera of BALB/c mice vaccinated with SOCS1-his recombinant protein. For detection of TANK in DF-1 cells, cell lysates were prepared using a non-denaturing lysis buffer (50 nM Tris-HCl, pH 8.0, 150 nM NaCl, 1% TritonX-100, 5 nM EDTA, 10% glycerol, 10 nM dithiothreitol, 1 × complete cocktail protease inhibitor). The cell lysates were boiled with 6 × SDS loading buffer for 10 min and fractionated by electrophoresis on 12% SDS-polyacrylamide gels, and resolved proteins were transferred onto polyvinylidene difluoride (PVDF) membranes. After blocking with 5% skimmed milk, the membranes were incubated with anti-TANK or anti-GAPDH antibody, followed by HRP-conjugated anti-Mouse secondary antibody. Blots were developed using an enhanced chemiluminesence (ECL) kit per the manufacturer's instruction.”

The authors apologize for these errors and state that this does not change the scientific conclusions of the article in any way. The original article has been updated.
