# Supplementary figures and images for: gga-miR-155 Enhances Type I Interferon Expression and Suppresses Infectious Burse Disease Virus Replication via Targeting SOCS1 and TANK
Source: Front Cell Infect Microbiol. 2018 Mar 7;8:55. doi: 10.3389/fcimb.2018.00055 (PMC5845882; doi:10.3389/fcimb.2018.00055)

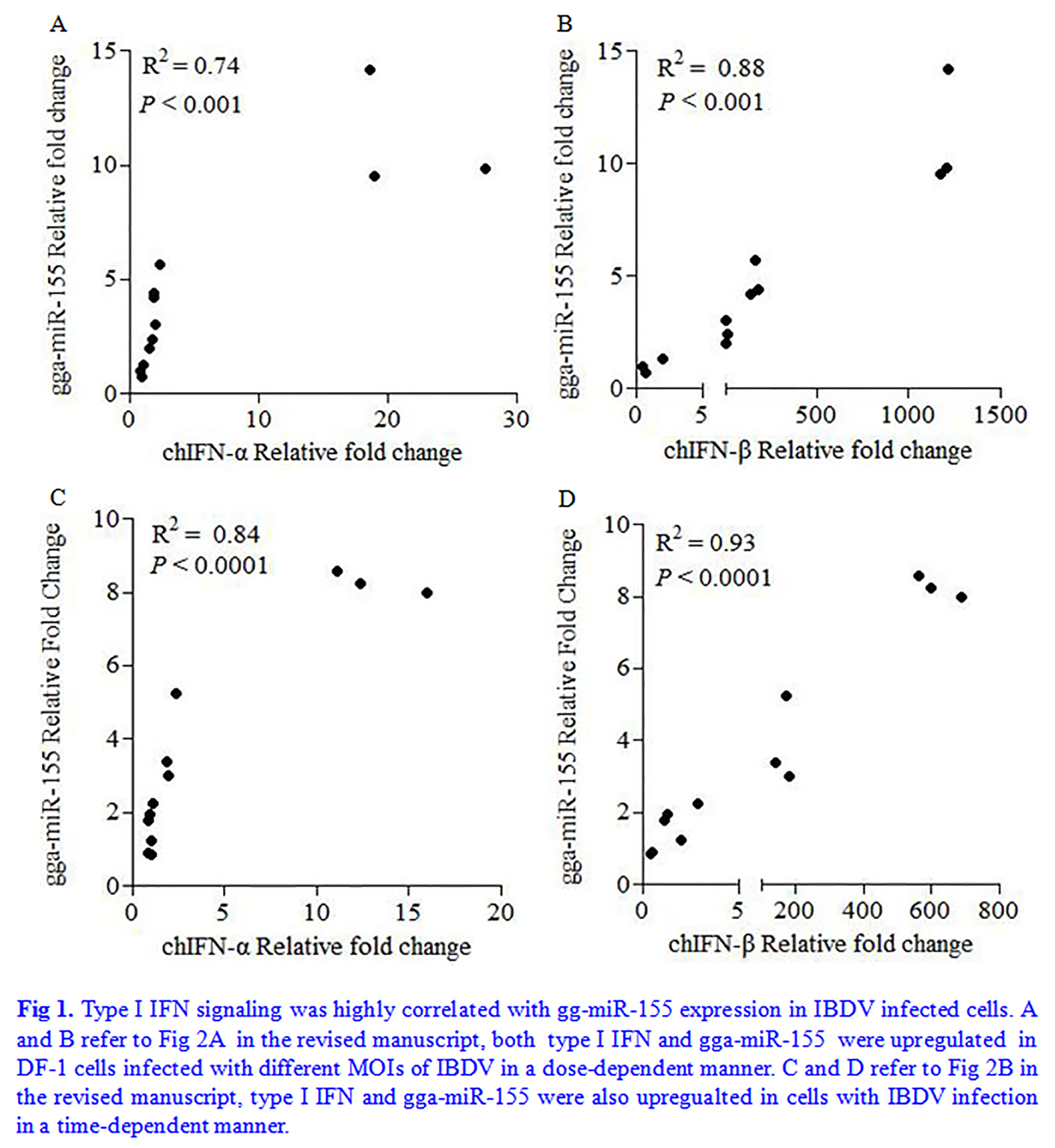

Supplement: Supplementary file 2 [file Image1.TIF]
